# *Taraxacum mongolicum* Ameliorates DNCB-Induced Atopic Dermatitis-like Symptoms in Mice by Regulating Oxidative Stress, Inflammation, MAPK, and JAK/STAT/TSLP Signaling Pathways

**DOI:** 10.3390/ijms26146601

**Published:** 2025-07-09

**Authors:** Wen-Ping Jiang, Hsi-Pin Hung, Jaung-Geng Lin, Ling-Huei Chang, Atsushi Inose, Guan-Jhong Huang

**Affiliations:** 1 School of Pharmacy, College of Pharmacy, China Medical University, Taichung 404, Taiwan; wpjiang@cmu.edu.tw; 2 School of Chinese Medicine, College of Chinese Medicine, China Medical University, Taichung 404, Taiwan; pin721.hwatou@gmail.com (H.-P.H.); jglin@mail.cmu.edu.tw (J.-G.L.); citizen5499@hotmail.com (L.-H.C.); 3 Chinese Medicine Research Center, China Medical University, Taichung 404, Taiwan; 4 Faculty of Pharmacy, Nihon Pharmaceutical University, Saitama 362-0806, Japan; ainose@nichiyaku.ac.jp; 5 Department of Food Nutrition and Healthy Biotechnology, Asia University, Taichung 413, Taiwan; 6 Department of Chinese Pharmaceutical Sciences and Chinese Medicine Resources, College of Chinese Medicine, China Medical University, Taichung 404, Taiwan

**Keywords:** atopic dermatitis, *Taraxacum mongolicum*, DNCB, cytokines, anti-inflammatory

## Abstract

Atopic dermatitis (AD) is a chronic inflammatory skin disease stemming from genetic susceptibility and environmental factors. It is characterized by immune dysregulation, increased mast cell activity, elevated levels of immunoglobulin E (IgE), and excessive proinflammatory mediator expression. These factors contribute to hallmark symptoms such as pruritus, erythema, and skin barrier dysfunction. In this study, we investigated the antioxidant and anti-inflammatory effects of *Taraxacum mongolicum* (WTM) water extract, as well as its skin barrier regulation and immune functions in AD. In the present study, we explored the therapeutic efficacy and underlying mechanisms of WTM in a BALB/c mouse model of AD induced by 2,4-dinitrochlorobenzene (DNCB). Mice were administered WTM orally or topically for 14 consecutive days. The results demonstrated that WTM treatment significantly alleviated clinical severity, showing reductions in skin lesion scores, epidermal thickness, mast cell infiltration, and scratching behavior, compared to the DNCB-treated group. Mechanistically, WTM reduced serum levels of IgE and proinflammatory cytokines (IL-4, IL-6, IL-1β, TNF-α, and IL-31) while suppressing the expression of the JAK/STAT/TSLP signaling pathway in skin tissues. Furthermore, WTM inhibited the TLR4/NF-κB and MAPK pathways and enhanced antioxidant defense by elevating superoxide dismutase (SOD), catalase, and glutathione peroxidase (GPx) activities. These findings indicate that WTM attenuates DNCB-induced AD progression in mice, likely through the dual modulation of inflammatory signaling and oxidative stress. These findings suggest that WTM may modulate the immune response and alleviate AD symptoms by inhibiting the TLR4/NF-κB, MAPK, and JAK/STAT/TSLP pathways.

## 1. Introduction

Atopic dermatitis (AD) is a prevalent, chronic, and recurrent inflammatory skin disorder affecting up to 20% of children and 10% of adults worldwide. In the United States, the condition affects approximately 18% of school-aged children and 7% of adults. While AD most commonly presents during infancy and childhood, adult-onset cases account for 20–25% of adults diagnosed with the disease [1,2]. Although non-fatal, AD is frequently associated with complications including pruritus, sleep disturbances, psychosocial distress, and depressive symptoms, all of which profoundly compromise quality of life. The pathogenesis of the condition is multifactorial, encompassing dysregulated local and systemic immune responses, impaired epidermal barrier function, environmental triggers, and neuropsychological interactions. Consequently, therapeutic management remains challenging. Current standard therapies comprise biologic agents, systemic immunosuppressants, and topical or oral corticosteroids [3]. Despite the latter being the most widely prescribed treatment for AD, their long-term utility is constrained by dose-limiting adverse effects, including cutaneous atrophy, telangiectasia, hyperpigmentation, and steroid-dependent dermatitis [4]. While corticosteroid-sparing agents such as cyclosporine, azathioprine, and methotrexate are employed as alternatives, their therapeutic outcomes remain suboptimal [5]. Although biologic therapies demonstrate clinical efficacy, prohibitive costs restrict accessibility for most AD patients, underscoring the urgent need for novel pharmacologic interventions that prioritize safety, minimal toxicity, and sustainable efficacy [6].

Thymic stromal lymphopoietin (TSLP) belongs to the IL-2 cytokine family; it is secreted by skin and other non-immune cells such as keratinocytes, fibroblasts, and smooth muscle cells [7]. When the skin is irritated by things like allergens, insect bites, or scratching, TSLP is released and sends signals to dendritic cells. These cells then activate T cells to start a type 2 immune response, which is typical in eczema and asthma [8]. Because of its key role in triggering inflammation, scientists now view TSLP as a major driver of allergic disease, which is why new targeted treatments are being actively developed [9]. TSLP has also been shown to activate mast cells and eosinophils, leading to the release of high levels of Th2-associated cytokines and thereby exacerbating allergic inflammation. Moreover, TSLP can directly stimulate sensory neurons, inducing itch responses that are independent of Th2 pathways. These findings collectively suggest that TSLP plays a pivotal role in both the initiation and perpetuation of AD by driving Th2-mediated inflammatory cascades, establishing it as a critical mediator in AD pathogenesis [10]. Importantly, TSLP expression is significantly upregulated in lesional skin of AD patients, correlating with disease severity and clinical symptoms such as erythema, edema, and chronic pruritus. This cytokine not only shapes the local immune microenvironment by modulating dendritic cell function but also facilitates the migration and activation of Th2 lymphocytes. The downstream effects include the elevated production of interleukins, such as IL-4, which further disrupt skin barrier integrity and enhance IgE production, compounding the allergic response [11]. In murine AD models, genetic deletion or the pharmacological inhibition of TSLP signaling has been shown to markedly reduce clinical symptoms, histological inflammation, and cytokine expression. These results provide compelling preclinical evidence for TSLP as a viable therapeutic target [12].

The Janus kinase/signal transducers and activators of transcription (JAK/STAT) signaling pathway is an emerging direction in the field of dermatology. This pathway is not only essential for the normal function of the immune system but may also be associated with the development of inflammatory skin diseases, especially psoriasis, AD, and melanoma [7]. It is a classical signal transduction pathway for many cytokines and growth factors. It plays a significant role in the signal amplification and interconnection of immunological pathways. JAK-STAT inhibitors alleviate AD by blocking JAK and STAT phosphorylation [8]. However, evidence regarding their clinical efficacy is still scarce [13]. Their dysregulation has been implicated in the pathogenesis of numerous inflammatory skin disorders, most notably AD, which is characterized by chronic relapsing eczema and a Th2-skewed immune profile. In AD, aberrant JAK/STAT pathway activation contributes to the overproduction of proinflammatory cytokines such as IL-4, IL-13, and IL-31, which are directly linked to barrier dysfunction, itch, and inflammation [14]. However, despite these promising results, the long-term efficacy and safety of JAK inhibitors remain incompletely understood, particularly concerning immunosuppression-related risks, rebound effects, and patient-specific variability. While the JAK/STAT pathway is a compelling therapeutic target, further clinical studies are essential to fully elucidate its role and optimize its use in AD treatment strategies. Beyond the JAK/STAT pathway, both NF-κB and MAPK signaling are critically implicated in the inflammatory process underlying AD. NF-κB activation leads to the transcription of inflammatory mediators, while MAPK signaling contributes by activating AP-1 [15]. Together, these pathways drive immune cell infiltration and cytokine overproduction. Their interconnection with JAK/STAT creates a complex signaling network that exacerbates disease severity. Understanding this crosstalk is crucial for identifying multi-targeted treatment approaches that may improve outcomes in chronic AD management.

*Taraxacum mongolicum* (dandelion) has long been used in traditional Asian medicine for managing infections such as bronchitis, tonsillitis, and urinary tract inflammation, largely owing to its reputed heat-clearing and anti-inflammatory effects [16]. Recent studies have confirmed that *T. mongolicum* water extracts exert protective effects against LPS-induced lung inflammation, primarily attributed to antioxidant constituents including caffeic, cichoric, and chlorogenic acids [17,18,19]. However, the underlying molecular mechanisms and immunoregulatory potential of *T. mongolicum* in systemic inflammatory models remain poorly characterized. This study aims to explore the immunomodulatory properties of a water extract of *T. mongolicum* (WTM) and delineate its mechanism of action using a DNCB-induced mouse model of inflammation. Our results demonstrated that WTM modulates immune responses by regulating the TLR4/NF-κB, MAPK, and JAK/STAT/TSLP signaling pathways, thereby suppressing inflammatory cascades. These findings provide novel mechanistic insight into the therapeutic potential of *T. mongolicum*, highlighting its promise as a complementary or alternative strategy for treating immune-related infectious diseases.

## 2. Results

### 2.1. Analysis of HPLC of WTM

**Figure 1:**
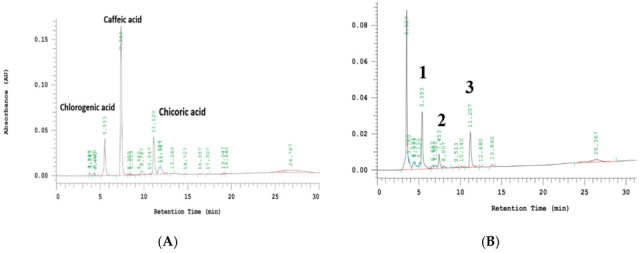
HPLC chromatographic analysis showing the standard marker (**A**) and profile of WTM (**B**). The peaks indicate 1. chlorogenic (5.39 min); 2. caffeic (7.45 min); and 3. cichoric acids (11.21 min). Red line represents the baseline in the HPLC analysis, which indicates the detector’s signal when no compounds are eluting. A stable baseline is essential for accurate measurement, as it serves as the reference point for detecting peaks. Black line shows the detection of compounds as they elute from the HPLC column. Each peak corresponds to a different compound, with the retention time (x-axis) helping in identification and the peak area/height (y-axis) relating to concentration.

A reliable high-performance liquid chromatography method coupled with photodiode array detection (HPLC-DAD) was established to assess WTM quality by constructing a comprehensive chromatographic fingerprint. The resulting HPLC chromatogram revealed the presence of three key marker compounds within the extract. As illustrated in Figure 1, these components were identified as chlorogenic (5.39 min), caffeic (7.45 min), and cichoric acids (11.21 min) based on their retention times and UV spectra. Quantitative analysis indicated that the respective concentrations of these compounds in WTM were 112.12 μg/mL for chlorogenic, 264.45 μg/mL for caffeic, and 783.76 μg/mL for cichoric acids.

### 2.2. Effect of DNCB on Disease Severity

**Figure 2:**
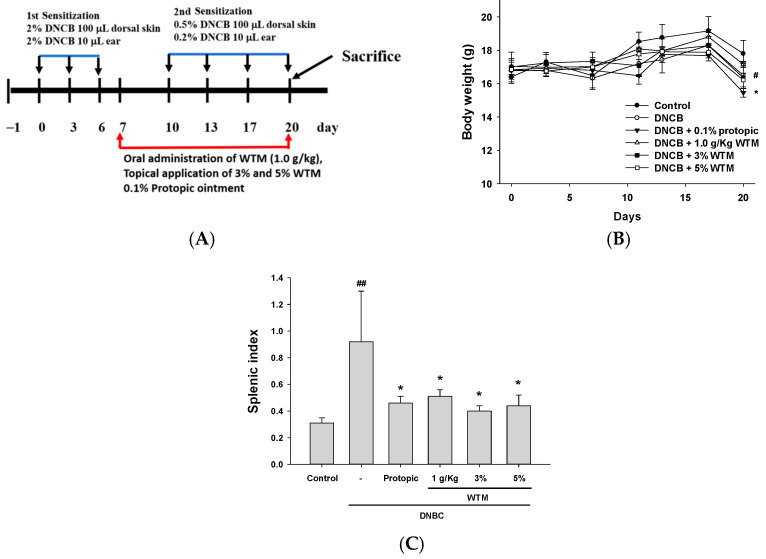
Therapeutic WTM administration effectively reduced dermatitis severity in the DNCB-induced mouse model of AD. The study design (**A**) involved routine measurements of body weight (**B**) and spleen index (**C**) across all experimental groups. All animals were sacrificed on day 20 for final analysis. Data represent mean ± SEM (n = 5). Significant differences are indicated as follows: ^#^ *p* < 0.05 and ^##^ *p* < 0.01 compared with control; * *p* < 0.05 compared with DNCB group.

The DNCB induction method used in this experiment is illustrated in Figure 2A. In the first phase, mice were sensitized with 2% DNCB, followed by repeated 0.2% or 0.5% topical applications during the second phase. Although all groups showed an increase in body weight over time, by day 20, a significant difference was observed between the control and DNCB groups (Figure 2B). It is well known that secondary lymphoid organs, such as the spleen, play a key role in regulating antigen-driven lymphocyte maturation [5]. The DNCB group exhibited a significant spleen weight and size increase compared to the control group. Notably, spleen enlargement was attenuated in the WTM-treated groups, with a statistically significant reduction compared to the DNCB group (Figure 2C). Collectively, these results indicate that WTM may modulate antigen-induced immune responses by ameliorating splenic hypertrophy and reducing overall disease severity.

### 2.3. WTM Treatment Was Evaluated Using a DNCB-Induced AD-like Skin Model in BALB/c Mice to Assess Its Anti-Dermatitic Effects

To evaluate the therapeutic potential of WTM against AD, we utilized a DNCB-induced AD-like skin lesion model in BALB/c mice. The dorsal hair was removed using scissors, and the skin was sensitized with 2% DNCB. WTM was administered orally (1.0 g/kg) or applied topically at 3% and 5% concentrations daily for 14 days (7–20 days). To enhance disease severity, 0.2% or 0.5% DNCB was topically applied every four days (days 10, 13, 17, and 20).

**Figure 3:**
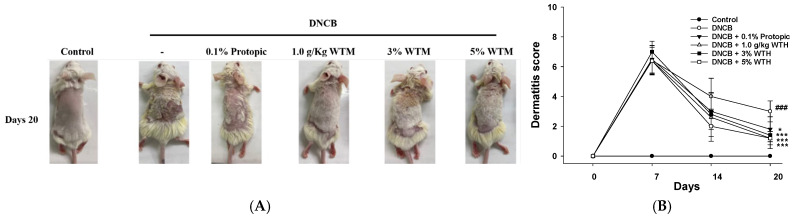

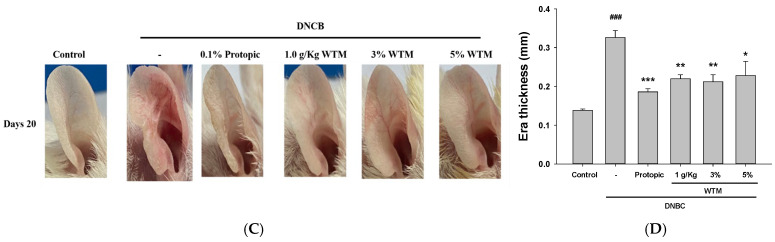
Effects of WTM on AD in DNCB-induced BALB/c mice. The dorsal skin and representative images were obtained at identical magnification (**A**). Dermatitis score (**B**). Representative images (**C**) and ear thickness (**D**) were measured on the final day, day 20 (n = 5). Data represent mean ± SEM (n = 5). Significant differences are indicated as follows: ^###^ *p* < 0.001 compared with control; * *p* < 0.05, ** *p* < 0.01, and *** *p* < 0.001 compared with DNCB group.

The DNCB-treated group showed visibly redder and more damaged skin compared to the control group, with significantly increased dermatitis scores. In contrast, oral and topical WTM treatments visibly reduced redness and lesions. The dermatitis scores significantly and dose-dependently decreased with WTM treatment (Figure 3A,B). Moreover, ear vessels in the DNCB group appeared congested and reddish, and ear thickness was markedly increased. WTM treatment significantly reduced ear thickness (Figure 3C,D). These findings demonstrate that both oral and topical WTM effectively improve clinical manifestations of AD in this murine model.

### 2.4. WTM Alleviates DNCB-Induced Scratching Behavior in Mice

**Figure 4:**
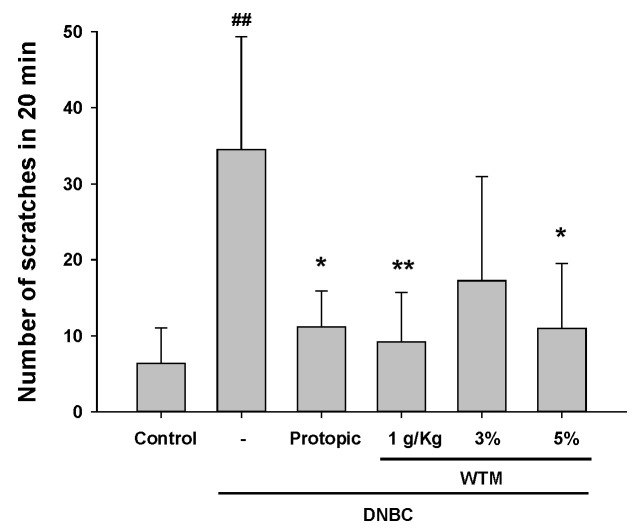
The effects of WTM on scratching frequency were evaluated in DNCB-induced mice. Scratching events were quantified over 20 min by analyzing video recordings. Data represent mean ± SEM (n = 5). Significant differences are indicated as follows: ^##^ *p* < 0.01 compared with control; * *p* < 0.05, and ** *p* < 0.01 compared with DNCB group.

Scratching behavior is a characteristic symptom of AD in both humans and murine models; it is often utilized as a reliable metric of disease severity [2]. To assess the antipruritic efficacy of WTM in the DNCB-induced AD model, we measured scratching time by observing the amount of time mice spent rubbing their dorsal skin and ears with their hind limbs. On the final day, quantitative analysis revealed that scratching time was significantly increased in DNCB-treated mice compared to controls. Treatment with WTM, whether administered orally or applied topically at 3% or 5%, significantly reduced scratching behavior (Figure 4). These findings support WTM’s potential as an effective antipruritic agent for AD management.

### 2.5. WTM Ameliorates DNCB-Induced Histological Changes and Mast Cell Infiltration

**Figure 5:**
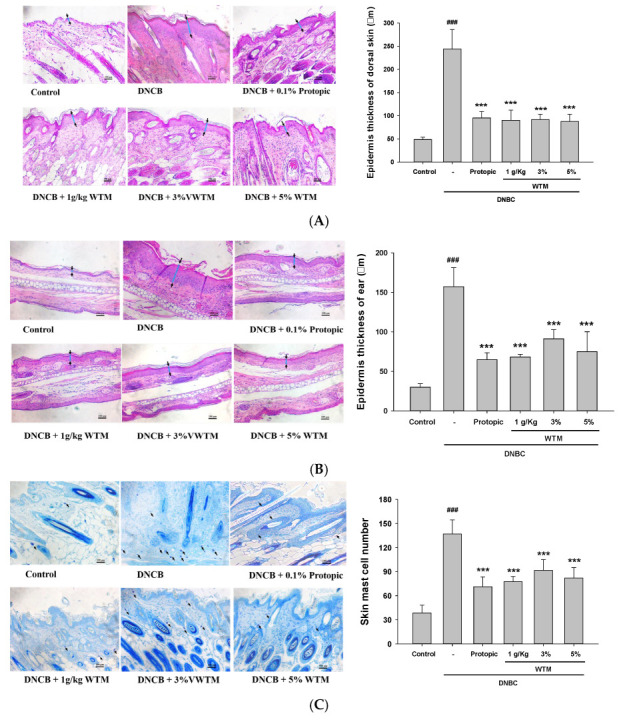

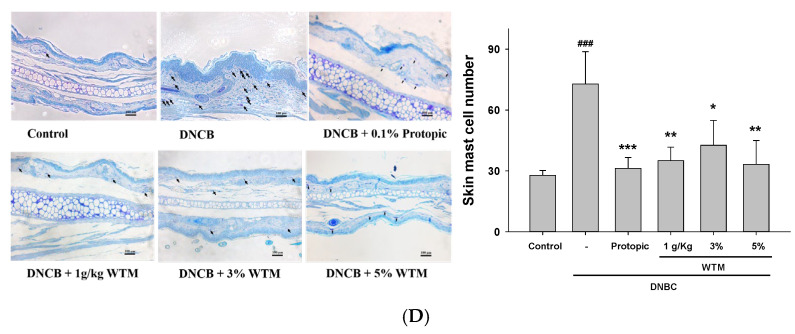
The effects of WTM on dermal and epidermal thickness, as well as mast cell infiltration, were histologically examined. Histopathological evaluation was performed via hematoxylin and eosin (H&E) (**A**,**B**) and toluidine blue staining (**C**,**D**) of AD-like dorsal and ear skin lesions (magnification ×200; scale bar: 100 µm). Dermal and epidermal thickness was quantitatively assessed from H&E-stained micrographs (**A**,**B**), while mast cells were identified and counted in toluidine blue-stained sections across five high-power fields using a Nikon microscope (Nikon Eclipse TS100; Nikon Corp., Tokyo, Japan). Data represent mean ± SEM (n = 5). Significant differences are indicated as follows: ^###^ *p* < 0.001 compared with control; * *p* < 0.05, ** *p* < 0.01, and *** *p* < 0.001 compared with DNCB group.

Histological alterations were assessed by examining epidermal hyperplasia and mast cell infiltration in dorsal skin and ear lesions using H&E and toluidine blue staining, respectively. Control group tissues displayed a thin epidermal layer, well-structured dermal collagen, and an absence of inflammatory infiltration. In contrast, the AD group showed pronounced epidermal and dermal thickening, hyperkeratosis, and inflammatory cell infiltration. Notably, both the oral administration and topical application (3% and 5%) of WTM significantly reduced epidermal and dermal thickness compared to untreated AD mice (Figure 5A,B), suggesting that WTM mitigates DNCB-induced histopathological changes.

Mast cell infiltration is a hallmark of AD, driving disease progression through the release of proinflammatory cytokines and mediators [5]. We further investigated WTM’s effect on mast cell accumulation and found that AD mice displayed severe mast cell infiltration in dorsal skin and ear tissues compared to controls. However, WTM treatment (oral or topical) significantly attenuated mast cell infiltration and reduced mast cell numbers (Figure 5C,D). Together, these findings demonstrate that WTM alleviates DNCB-induced epidermal hyperplasia, dermal thickening, and mast cell recruitment.

### 2.6. WTM Administration Attenuates Oxidative Stress in DNCB-Induced Mice

**Figure 6:**
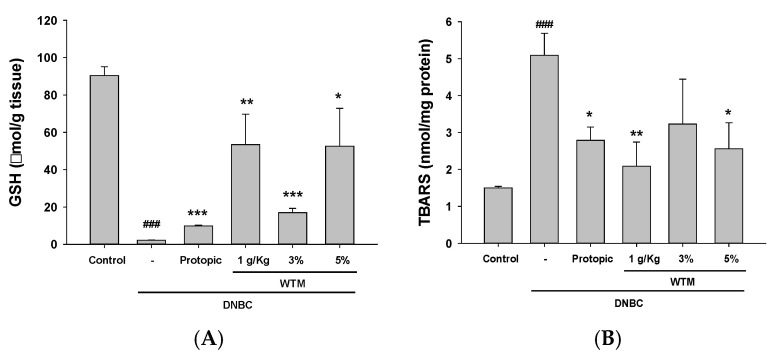
WTM alleviates oxidative stress in DNCB-induced mice, as evidenced by the reduced MDA levels (**A**) and elevated GSH levels (**B**). To assess oxidative stress, MDA and GSH levels were measured in skin tissue homogenates. Data represent mean ± SEM (n = 5). Significant differences are indicated as follows: ^###^ *p* < 0.001 compared with control; * *p* < 0.05, ** *p* < 0.01, and *** *p* < 0.001 compared with DNCB group.

WTM administration attenuated oxidative stress in DNCB-induced mice. Given the established relationship between oxidative stress and AD, we evaluated the in vivo effects of WTM on oxidative biomarkers. As shown in Figure 6A,B, DNCB exposure significantly decreased glutathione (GSH) levels while increasing malondialdehyde (MDA) concentrations. WTM treatment markedly restored GSH levels and reduced MDA accumulation, suggesting that it effectively mitigates oxidative stress in DNCB-induced AD.

### 2.7. The Therapeutic Effect of WTM on DNCB-Induced Atopic Dermatitis May Be Attributed to Its Ability to Regulate IgE Production and Inhibit the Expression of Proinflammatory Cytokines

**Figure 7:**
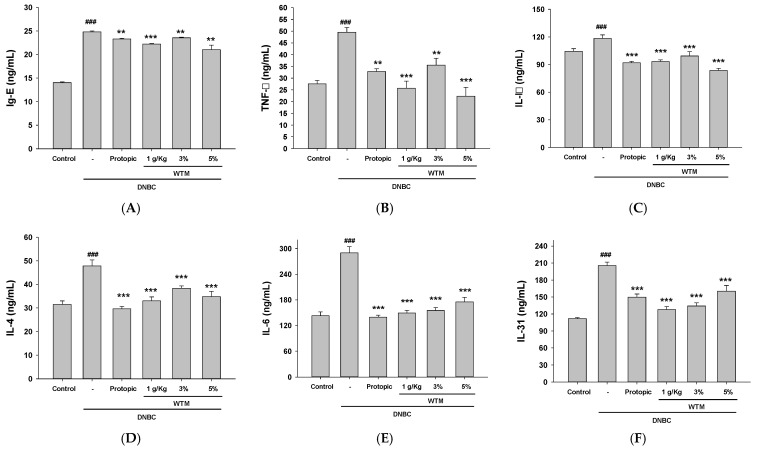
The administration of WTM inhibited the serum levels of IgE (**A**), TNF-α (**B**), IL-1β (**C**), IL-4 (**D**), IL-6 (**E**), and IL-31 (**F**) in a DNCB-induced mouse model of AD. Serum concentrations of these markers were measured using ELISA kits (**A**–**F**). Significant differences are indicated as follows: ^###^ *p* < 0.001 compared with control; ** *p* < 0.01, and *** *p* < 0.001 compared with DNCB group.

The involvement of the immune response in AD pathogenesis has been well established, with IgE serving as a principal indicator of allergic reactions [5]. To assess the immunoregulatory effect of WTM, serum concentrations of IgE, IL-4, IL-6, IL-1β, TNF-α, and IL-31 were measured by an ELISA. DNCB stimulation significantly increased serum IgE levels in AD mice compared to controls. In contrast, WTM treatment effectively reduced IgE levels (Figure 7A). Furthermore, the expression of TNF-α, IL-1β, IL-4, IL-6, and IL-31 in skin lesions was markedly elevated following DNCB exposure but significantly decreased after WTM administration (Figure 7B–F). These results suggest that WTM mitigates systemic and local allergic inflammation in DNCB-induced AD mice.

### 2.8. WTM Reduced Inflammation and Inhibited Both NF-κB and MAPK Signaling Pathways in DNCB-Induced Mice

**Figure 8:**
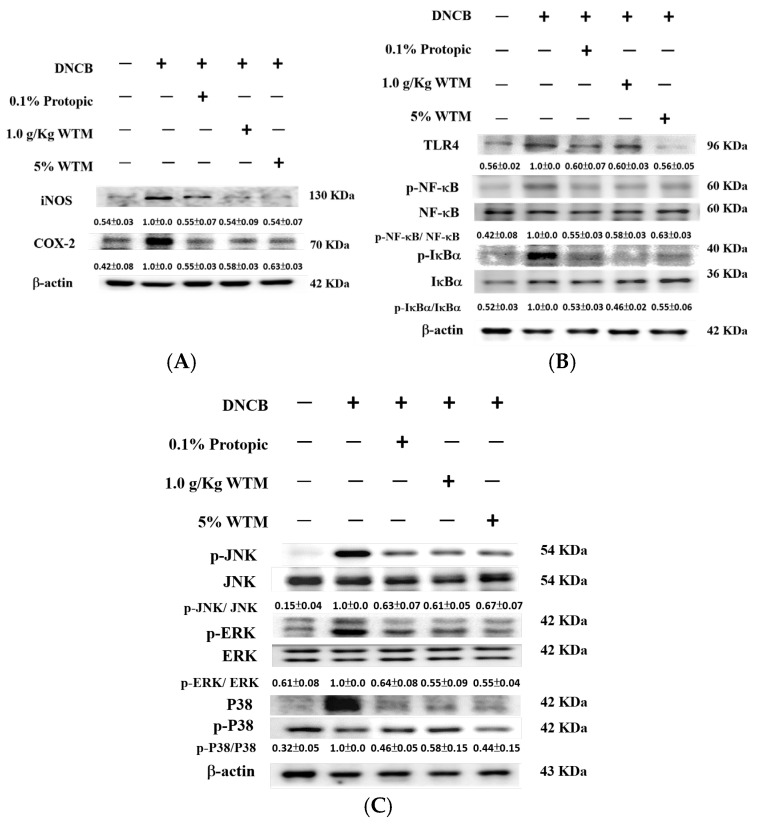
WTM suppressed the DNCB-induced protein expression of iNOS, COX-2 (**A**), TLR4, NF-κB, IκB-α (**B**), and phosphorylated MAPKs (**C**) in mouse skin tissues. The protein expression levels of the target enzymes were assessed in skin tissue homogenates using Western blotting after DNCB exposure. Results are expressed as mean ± SEM. Densitometric analysis was performed, and protein levels were normalized to β-actin, which served as the internal loading control. Data are presented as fold changes relative to the control group.

Inflammatory responses involve intricate molecular mechanisms. As shown in Figure 8A, when administered orally or topically at a 5% concentration, WTM demonstrated a robust ability to suppress iNOS and COX-2 expression in inflamed skin tissues. Notably, its efficacy was comparable to that of the standard treatment, 0.1% Protopic, which also substantially reduced the expression of these inflammatory markers in the AD model.

Toll-like receptors (TLRs) function as key immune sensors that recognize pathogen-associated molecular patterns in DNCB-induced AD, thereby initiating inflammatory signaling cascades [20]. Western blot analysis revealed TLR4 activation following DNCB injection (Figure 8B). Notably, when administered orally or topically (5%), WTM significantly inhibited TLR4 upregulation, highlighting its modulatory role in the TLR4-mediated inflammatory response. Moreover, NF-κB pathway activation, crucial for initiating inflammatory cascades, was observed after DNCB administration, as indicated by increased levels of phosphorylated NF-κB (p-NF-κB) and phosphorylated IκBα (p-IκBα). WTM treatment notably suppressed the expression of p-NF-κB and p-IκBα in skin tissues, suggesting its regulatory effect on TLR4/NF-κB signaling in acute inflammation (Figure 8B).

The initiation of the inflammatory response is mediated by signal transduction pathways, particularly those involving mitogen-activated protein kinases (MAPKs) and transcription factors. MAPK pathway activation has been implicated in the pathogenesis of various inflammatory diseases [21]. As shown in Figure 7C, DNCB exposure significantly increased the phosphorylation levels of JNK, ERK, and p38 in skin tissues, indicating MAPK pathway activation. Notably, oral and 5% topical WTM treatments effectively suppressed these elevations (Figure 8C). Changes in MAPK expression were evident at the phosphorylation level and without affecting total protein levels, suggesting that WTM reduces phosphorylated MAPK proteins in DNCB-induced AD.

### 2.9. WTM Enhances Antioxidant Defense Mechanisms in Skin Tissues to Alleviate DNCB-Induced AD

**Figure 9:**
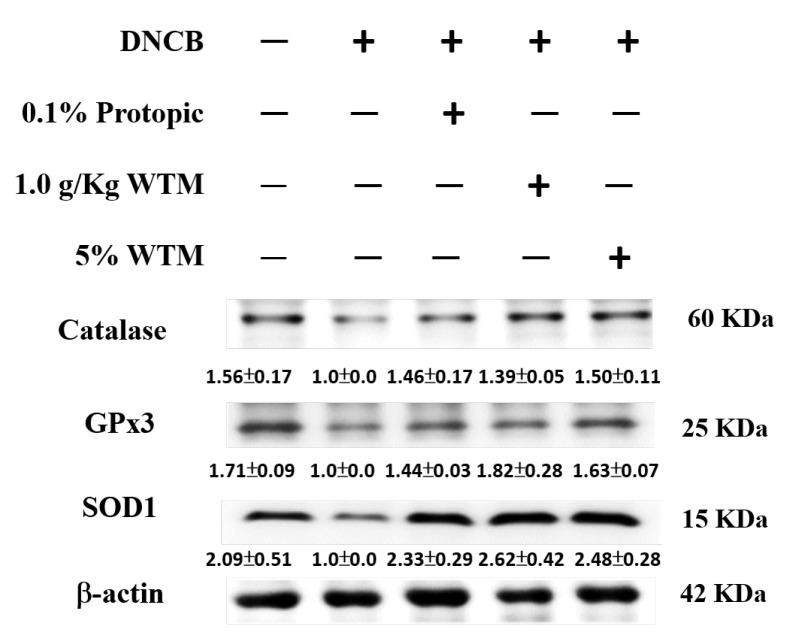
WTM modulates DNCB-induced alterations in the antioxidative enzyme (catalase, SOD1, and GPx3) expression in skin tissues. The protein expression levels of the target enzymes were assessed in skin tissue homogenates using Western blotting after DNCB exposure. Results are expressed as mean ± SEM. Densitometric analysis was performed, and protein levels were normalized to β-actin, which served as the internal loading control. Data are presented as fold changes relative to the control group.

Oxidative stress contributes to tissue damage and inflammation, exacerbating disease progression [10]. As illustrated in Figure 9, DNCB exposure significantly reduced the expression of key antioxidant enzymes—catalase, superoxide dismutase (SOD) and glutathione peroxidase (GPx)—in skin tissues. Notably, both oral and 5% topical WTM application markedly restored these antioxidant protein levels toward baseline values, suggesting its protective role against oxidative damage.

### 2.10. WTM Attenuates Dysregulation of the JAK-STAT3-TSLP Signaling Pathway in DNCB-Induced AD

**Figure 10:**
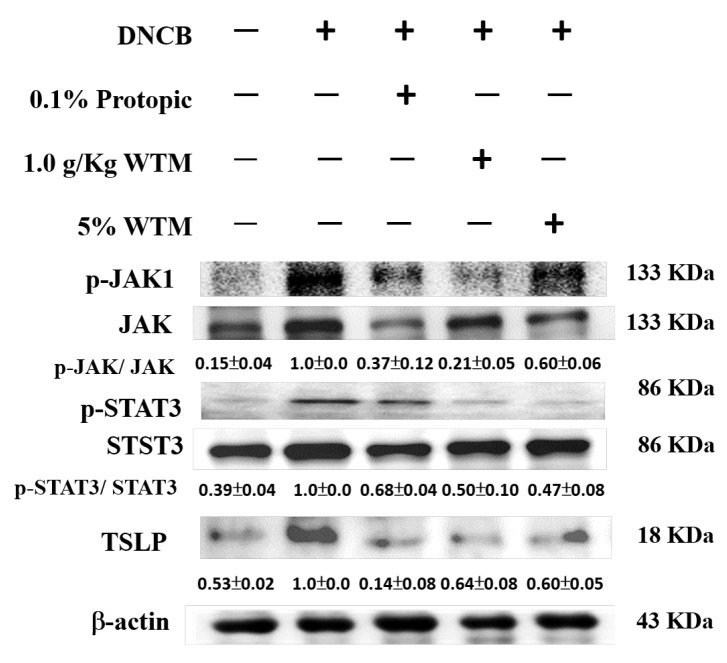
WTM significantly attenuated the expression of the JAK-STAT3-TSLP signaling axis following DNCB exposure. The protein expression levels of the target enzymes were assessed in skin tissue homogenates using Western blotting after DNCB exposure. Results are expressed as mean ± SEM. Densitometric analysis was performed, and protein levels were normalized to β-actin, which served as the internal loading control. Data are presented as fold changes relative to the control group.

The JAK-STAT3-TSLP pathway is critically involved in allergic diseases such as AD [12]. As illustrated in Figure 10, DNCB exposure led to elevated expression levels of phosphorylated JAK1 (p-JAK1), phosphorylated STAT3 (p-STAT3), and thymic stromal lymphopoietin (TSLP) in mouse skin tissues. Notably, both oral and 5% topical WTM treatments significantly suppressed the expression of these proteins compared to the DNCB group. These findings suggest that WTM mitigates DNCB-induced inflammation by modulating the JAK-STAT3-TSLP axis.

### 2.11. Mechanism Description

**Figure 11:**
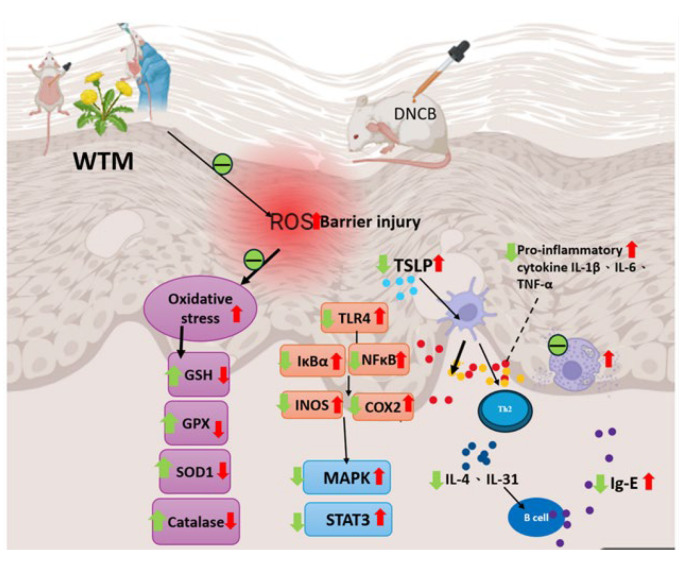
A diagrammatic depiction of WTM’s mechanism in treating AD. Red arrows are used to indicate an increase, while green arrows indicate a decrease. ROS: reactive oxygen species; GSH: glutathione; MAPK: mitogen-activated protein kinase; NF-κB: nuclear factor of κB; IκB-α: NF kappa B inhibitor α; SOD1: Cu/Zn superoxide dismutase 1; GSH: glutathione; STAT3: signal transducer and activator of transcription 3; TLR-4: toll-like receptor 4; INOS: inducible nitric oxide synthase; COX-2: cyclooxygenase-2; TNF-α: tumor necrosis factor-α; IL-1ß: interleukin-1β; IL-6: interleukin-6; IL-4: interleukin-4; IL-31: interleukin-31.

Based on the observed data, WTM may improve AD symptoms by modulating the Th1/Th2 immune response and reducing IgG accumulation at lesion sites. This is further supported by its ability to inhibit MAPK and JAK/STAT/TSLP pathway activation, decrease IL-4, IL-6, IL-1β, TNF-α, and IL-31 production, and alleviate oxidative stress and inflammation (Figure 11).

## 3. Discussion

AD, often called eczema, is a long-term skin condition linked to family history and allergies. Patients experience extremely dry skin and uncontrollable itching. Scientists believe AD develops when two key systems fail: the immune system overreacts to triggers, and the skin’s protective layer breaks down [22]. These problems result from a mix of genes, environmental factors, and immune miscommunication. AD usually starts in babies and is becoming more common worldwide. The condition worsens when specific immune cells (Th2 cells) release signaling proteins (cytokines) that make B-cells produce too much IgE, an antibody involved in allergies. These cytokines also block other immune cells (Th1 cells), creating an imbalance [4]. High IgE levels then attach to mast cells and basophils, which release chemicals causing redness, swelling, and itching—the hallmarks of AD flare-ups [23]. The DNCB-induced mouse model in our study displayed hallmark features of AD, including increased skin severity scores and ear thickness, as well as histological evidence of epidermal thickening and elevated mast cell infiltration. In parallel, a significant upregulation of IL-4, IL-13, and JAK/STAT signaling proteins was observed, confirming the Th2-skewed immune response. Collectively, these results validate the model’s relevance to AD pathogenesis.

AD is initiated by sensitization to environmental antigens, microbial infections, and psychological stress, often in the context of compromised epidermal barrier function. Impaired skin barriers allow for the increased penetration of microbial components, which in turn activate innate immune receptors and trigger the proinflammatory signaling pathways that contribute to allergic sensitization [15]. TSLP has emerged as a crucial trigger in the onset of allergic diseases, owing to its potent capacity to drive naïve CD4^+^ T cells toward a Th2-dominant phenotype. The resulting cytokine profile—marked by high levels of IL-4, IL-5, IL-13, and TNF-α and low levels of IFN-γ and IL-10—creates a highly inflammatory milieu that fuels the progression of allergic conditions [11]. This skewing of the cytokine profile from a Th1 to Th2 dominance further exacerbates allergic symptoms. Experimental studies in TSLP-deficient mice have demonstrated attenuated allergic responses, characterized by reduced eosinophilic infiltration, diminished Th2 cytokine production, and lower serum IgE levels. In contrast, mice expressing a skin-specific, inducible TSLP transgene develop eczematous skin lesions, indicating TSLP’s causal role in AD pathogenesis [15]. Moreover, TSLP is markedly overexpressed in the lesional skin of AD patients. The interaction between TSLP-producing epithelial and dendritic cells in the skin or respiratory tract is thought to be central to the initiation and maintenance of Th2-dominant allergic inflammation. TSLP signaling enhances Th2 responses by activating the FcεRI pathway and upregulating Th2-attracting chemokines such as TARC/CCL17 [24]. Previous studies have also shown that mast cells contribute to TSLP production via intracellular calcium signaling, caspase-1 activation, and NF-κB pathways. Given the role of FcεRI-bearing mast cells in AD, therapeutic strategies that target these cells or suppress TSLP expression may be beneficial [25]. The present study observed a significant reduction in TSLP expression in the skin of DNCB-induced AD mice following WTM treatment. This TSLP level modulation may be associated with the anti-inflammatory and -allergic outcomes observed, supporting the possibility that WTM exerts its effects through the partial involvement of the TSLP signaling pathway.

AD is initiated by sensitization to environmental antigens, infections, and psychological stressors through a compromised epidermal barrier [26]. The enhanced penetration of microbial components across dysfunctional skin barriers activates innate immune receptors (e.g., TLRs), triggering proinflammatory signaling that exacerbates sensitization. Recent studies propose TSLP as a pivotal switch in allergic inflammation, driving the differentiation of naive CD4^+^ T cells into proinflammatory Th2 subsets characterized by elevated IL-4, IL-5, IL-13, and TNF-α secretion, alongside suppressed IFN-γ and IL-10 production [27]. This Th2-skewed cytokine milieu perpetuates allergic symptoms. The genetic ablation of TSLP in murine models attenuates allergic responses, as evidenced by reduced eosinophil infiltration, Th2 cytokine levels, and serum IgE. Conversely, transgenic mice overexpressing TSLP in the skin develop eczematous lesions mirroring human AD [12]. Clinically, TSLP is markedly upregulated in AD lesions and facilitates crosstalk between epithelial and dendritic cells (DCs), amplifying Th2 polarization via FcεRI-dependent pathways and TARC/CCL17 chemokine induction. Intriguingly, TSLP expression in mast cells is regulated by intracellular calcium, caspase-1, and NF-κB, implicating mast cell desensitization as a therapeutic target [28]. In this study, WTM significantly suppressed TSLP levels in DNFB-induced AD mice, suggesting that its anti-AD efficacy may stem from TSLP downregulation.

The acute phase of AD is characterized by the overexpression of Th2 cytokines, such as IL-4, IL-5, and IL-10, which stimulate elevated IgE production [23,24]. Conversely, during the chronic phase, Th1-derived cytokines, including IFN-γ, along with proinflammatory mediators such as IL-6 and TNF-α, are markedly elevated. In our study, treatment with WTM demonstrated a notable ability to suppress both Th1 and Th2 cytokine expression, indicating efficacy in alleviating inflammation associated with both acute and chronic AD lesions [29]. More specifically, WTM significantly inhibited IL-4, IL-6, IL-1β, TNF-α, IL-31, iNOS, and COX-2 expression levels in skin tissue. COX-2, an inducible isoform of cyclooxygenase, plays a pivotal role in allergic responses and mast cell-mediated inflammation. As inflammatory stimuli and endotoxins can upregulate COX-2 expression, suppressing COX-2 and related inflammatory mediators represents a promising strategy for anti-allergic drug development. WTM effectively reduced the expression of TNF-α, iNOS, and COX-2, suggesting its potential as a therapeutic agent for mast cell-dependent allergic inflammation.

Numerous investigations have demonstrated that immune dysregulation, inflammation, and oxidative stress are intricately linked to multiple intracellular signaling pathways in AD [30]. Among these, the TLR4/NF-κB signaling axis plays a pivotal role. NF-κB, a key inflammatory transcription factor, is consistently upregulated in AD models. TLR4, an essential pattern recognition receptor of the innate immune system, detects external pathogenic stimuli and triggers downstream NF-κB activation. This cascade ultimately regulates proinflammatory gene transcription. Upon stimulation by cytokines such as TNF-α, IL-1, and IL-6, IκB kinases (IKKs) phosphorylate IκBα, leading to its ubiquitination and proteasomal degradation, thereby releasing NF-κB to translocate into the nucleus [25]. Activated NF-κB subsequently enhances the expression of COX-2, iNOS, and various inflammatory mediators. Mechanistic analysis revealed that WTM markedly suppressed TLR4 expression, along with the phosphorylation of NF-κB and IκBα, in the DNCB-induced AD mouse model. This suggests that WTM mediates its anti-inflammatory effects, at least in part, through inhibiting the TLR4/NF-κB signaling cascade.

Targeting the MAPK signaling pathway represents a promising therapeutic strategy due to its critical involvement in inflammatory disease pathogenesis. In particular, the topical inhibition of p38 MAPK has been demonstrated to attenuate severe inflammation in the dermis [31]. Upon stimulation, proinflammatory cytokines such as TNF-α and IL-6 bind to their respective receptors, initiating a phosphorylation cascade that activates p38 and JNK, thereby enhancing cytokine production and perpetuating an inflammatory loop [32]. Our findings indicate that WTM effectively suppresses inflammation and modulates immune responses through inhibiting MAPK signaling. Nonetheless, further clinical trials are warranted to verify the anti-AD potential of WTM in human subjects.

Excessive impairment of the skin’s antioxidant defense system accelerates AD progression. Inflammatory responses stimulate cytokine secretion, promoting the generation of reactive oxygen species (ROS) in immune cells and leading to oxidative stress [33]. Persistent ROS production and the accumulation of oxidative damage are associated with immune dysregulation and heightened susceptibility to disorders such as AD. Elevated ROS levels drive the infiltration of inflammatory cells and contribute to tissue damage. Moreover, oxidative stress in AD adversely affects lipids, proteins, and DNA, with lipid peroxidation serving as a potential endogenous danger signal implicated in the pathogenesis of AD. In support of this hypothesis, we quantified MDA and GSH levels in skin tissue, as these markers reflect lipid peroxidation and oxidative stress [34]. In the WTM-treated group, MDA levels showed a significant reduction, contrasting with the DNCB control group. Lipid peroxidation is increasingly recognized as a pathological contributor to inflammation. Furthermore, allergic reactions compromise antioxidant enzymes, exacerbating oxidative damage [35]. To evaluate antioxidative efficacy, we measured the activities of catalase, SOD, and GPx and observed significantly enhanced expressions of these enzymes in the WTM group. These findings suggest that WTM exerts a balneotherapeutic effect by boosting antioxidant enzyme activities, potentially mitigating DNCB-induced AD-like inflammation in mice. However, future research must clarify the exact molecular mechanisms behind these antioxidant effects, especially regarding redox-sensitive immune signaling pathways.

TSLP, a cytokine structurally related to IL-7, is produced by a variety of cell types and influences a diverse range of target cells, including immune cells like B cells, T cells, DCs, eosinophils, and NK cells, as well as non-immune cells such as smooth muscle and tumor cells [8,9]. The multifunctional nature of TSLP has prompted extensive investigation into its signaling pathways. Notably, its regulatory roles in allergic inflammation, particularly in AD and asthma, have become prominent research areas [11]. The JAK/STAT pathway, a classical signaling cascade associated with cell survival and immunosuppression, plays a critical role in mediating inflammatory responses. Numerous cytokines, such as TSLP, IL-10, IL-6, and IFN-γ, signal through the JAK2/STAT3 axis, with JAK2 acting as a key mediator of intracellular signal transduction [12]. Although it is well established that TSLP activates STATs in various cell types, the precise mechanisms involving JAK kinases remain poorly understood. Notably, the specific JAK and STAT subtypes activated by TSLP signaling appear to vary depending on the cell type and species.

The JAK/STAT pathway is a central signaling cascade that mediates the effects of numerous cytokines and plays a pivotal role in the pathogenesis of inflammatory and autoimmune diseases [8]. In AD, this pathway is particularly crucial for orchestrating immune responses, including its Th2-dominant immune activation. JAK1 and JAK2 function as proximal signaling molecules that transduce extracellular cytokine signals—such as those from IL-4, IL-13, IL-6, and IL-33—across membrane receptors, leading to the activation of downstream STAT proteins. Among these, STAT1 and STAT2 are primarily activated by IFN-γ and other Th1-associated cytokines, contributing to persistent skin inflammation and tissue damage during the chronic phase of AD. STAT3 and its phosphorylated form (P-STAT3) are activated by proinflammatory cytokines such as IL-6 and IL-23 [13]. This activation compromises skin barrier integrity and amplifies Th17-mediated inflammation. Furthermore, STAT3 also participates in Th2-related signaling pathways, including those involving TSLP and IL-33, positioning it as a key integrative molecule that links Th2 and Th17 immune responses in AD [35]. STAT6 is another critical transcription factor in this context, mediating IL-4 and IL-13 signaling and regulating hallmark features of the Th2 response, such as IgE production, mast cell activation, and eosinophil recruitment. In this study, we examined the expression and activation profiles of JAK/STAT pathway components to better understand their roles in the immunopathology of AD. Notably, elevated levels of activated STAT proteins—particularly P-STAT3—were observed, indicating cytokine signaling hyperactivation and its association with chronic inflammation and tissue injury in AD. These findings underscore the potential of targeting specific JAK/STAT molecules as a therapeutic strategy to mitigate inflammation and prevent disease progression in patients with AD.

The *Taraxacum* genus has been traditionally recognized for its therapeutic properties, including heat clearance, detoxification, swelling reduction, nodule dissipation, and diuretic effects [36]. A clinical trial (NCT00442091) registered at ClinicalTrials.gov and conducted by Odense University Hospital investigated the potential therapeutic effect of dandelion juice on dyshidrotic hand eczema. The primary organic acid constituents of *T. mongolicum* exhibit significant anti-inflammatory properties. These organic acids have been shown to modulate the TLR4/NF-κB signaling pathway, thereby mitigating LPS-induced pathological tissue damage and potentially offering a therapeutic avenue for acute tracheobronchitis [37]. Notably, the organic acid fraction of *T. mongolicum* includes compounds such as chlorogenic, caffeic, and cichoric acids, which are known for their anti-inflammatory and -oxidant activities.

Chlorogenic acid, also referred to as caffeoylquinic acid, is a polyphenolic compound that is abundantly present in various traditional Chinese medicinal herbs, often dubbed “plant gold” due to its diverse therapeutic potential. Chlorogenic acid exhibits diverse bioactivities, such as modulating immune responses, reducing oxidative stress, and attenuating inflammatory processes [38]. It has demonstrated therapeutic efficacy in systemic lupus erythematosus, colitis, and rheumatoid arthritis. Experimental findings show that chlorogenic acid significantly attenuates dermatitis severity, spleen index, epidermal hyperplasia, mast cell infiltration, and dermal fibrosis. Furthermore, chlorogenic acid reverses DNCB-induced elevations in serum IgE, histamine, TNF-α, IL-1β, IL-6, and IL-8 levels. Mechanistically, chlorogenic acid exerts its anti-inflammatory effects via PI3K/Akt and NF-κB signaling pathway suppression [5]. The precise function and underlying mechanisms of caffeic acid in AD remain to be elucidated. Arabica coffee extract, which contains chlorogenic acid and trace levels of caffeic acid, has demonstrated anti-inflammatory and skin barrier-restorative properties in a DNCB-induced mouse model of AD. The topical administration of Arabica coffee extract significantly alleviated erythema and reduced proinflammatory cytokine levels in affected skin areas [38]. Additionally, Arabica coffee extract restored barrier integrity and suppressed immune cell infiltration. Cichoric acid, a naturally occurring polyphenolic compound, is predominantly found in plants such as *Cichorium intybus* and *Echinacea*. As a hydroxycinnamic and caffeic acid derivative, cichoric acid has garnered attention for its diverse biological activities, including antioxidant regulation, anti-aging effects, and protection against digestive system disorders [39]. It has been widely utilized in pharmaceuticals, dietary supplements, and functional foods. Pharmacologically, cichoric acid contributes to the regulation of glucose and lipid metabolism and exhibits anti-inflammatory, -oxidative, and -aging properties. Consequently, it is commonly employed as an immune-enhancing agent in the United States and Europe [40]. Recent studies have demonstrated that cichoric acid mitigates NLRP3-mediated pyroptosis in acute lung injury models by inhibiting ROS-induced mitochondrial damage [41]. Additional research indicates that cichoric acid induces ROS generation in 3T3-L1 preadipocytes, modulating PI3K/Akt and MAPK signaling and leading to mitochondrial dysfunction, caspase-3 activation, PARP cleavage, and subsequent apoptosis [42].

## 4. Materials and Methods

### 4.1. Chemicals and Reagents

DNCB was obtained from Sigma-Aldrich Chemical Co. (St. Louis, MO, USA). The BCA protein assay kit was purchased from Thermo Fisher Scientific (Waltham, MA, USA). Tacrolimus ointment 0.1% (Protopic 0.1%) was acquired from Fujisawa Healthcare Inc. (Fujisawa, Japan). Primary antibodies against COX-2, p-JNK, catalase, SOD1, GPx3, and TLR4 were obtained from GeneTex (San Antonio, TX, USA). Antibodies for JNK, p-ERK, ERK, p-p38, p-JAK1, JAK1, STAT3, p-STAT3, and TSLP were sourced from Cell Signaling Technology (Beverly, MA, USA). Additionally, antibodies against iNOS, NF-κB, p-NF-κB, IκBα, p-IκBα, p38, and β-actin were purchased from Abcam (Cambridge, UK).

### 4.2. Material Sources

*T. mongolicum* (batch no. 202401) was acquired from Kang Li Drug Store (Taichung, China). Identification and authentication were performed by Wen-Ping Jiang (China Medical University). For extraction, 0.6 kg of dried *T. mongolicum* was soaked in ten volumes of distilled water for 1 h and then decocted twice for 30 min at boiling temperature. The decoctions were pooled and centrifuged at 3000 rpm for 5 min, and the supernatant was vacuum-dried at 55 °C. The extraction yielded 21.73% of dried powder relative to the original dry mass. The resulting WTM powder was dissolved in distilled water and stored at 4 °C. Animal dosages were calculated based on the human equivalent dose of 1 g/kg (Diallo et al. [43]).

### 4.3. HPLC Analysis of WHM

To qualitatively analyze WHM, an HPLC equipped with a photodiode array detector (Hitachi L-5000 series, Tokyo, Japan) was utilized. A 10 μL aliquot of extract was injected into a TSK-GEL ODS-80TM column (250 × 4.6 mm, 5 μm, Tosoh Co., Ltd., Tokyo, Japan), and chromatographic separation was monitored at 280 nm. The system was operated with a quaternary pump, autosampler, and column oven to ensure stability. A mobile phase gradient comprising 0.1% phosphoric acid (A) and acetonitrile (B) was optimized to enhance analyte resolution. The stepwise increase in B was as follows: 20% (0–5 min), 27% (5–12 min), 32% (12–14 min), 34% (14–17 min), 37% (17–27 min), and 45% (27–35 min). Reference standards of chlorogenic, caffeic, and chicoric acids were carefully prepared to ensure the accurate identification of WHM components.

### 4.4. Animals

Before experimental procedures, 7-week-old female BALB/c mice purchased from BioLASCO (Taipei, Taiwan) were allowed to acclimate to the laboratory environment for one week to minimize stress-related variables. Mice were housed under standard conditions (12 h light/dark cycle) with unlimited access to food and water. All animal procedures received prior approval from the Institutional Animal Care and Use Committee of China Medical University (Approval No. CMUIACUC-2023-399) and were conducted following institutional and national guidelines.

### 4.5. AD Models and Drug Treatment

To establish the atopic dermatitis model, BALB/c mice were randomly divided into five experimental groups (n = 5), including a healthy control group (no DNCB exposure), an oral treatment group, and three groups receiving topical interventions. AD-like symptoms were induced by applying 100 μL of 2% DNCB solution (in acetone/olive oil, 3:1) to the shaved dorsal skin and 10 μL to each ear lobe. The treatment regimen included four interventions: oral administration of WTM (1.0 g/kg; 20 mg/per mice), topical application of 100 μL 3% (12 mg/cm^2^) (stock solution 0.06 g/2 mL) and 100 μL 5% WTM (20 mg/cm^2^) (stock solution 0.1 g/2 mL), and 0.1% Protopic ointment (20 mg/cm^2^). The experimental protocol was adapted and modified from a previous study [44]. Twenty-four hours before DNCB sensitization, the hair was removed from a 2 × 2 cm^2^ area of the dorsal skin using an electric shaver, followed by depilatory cream. During the sensitization phase, on days 0, 3, and 6, 100 μL of 2% DNCB solution was applied to the shaved area and both inner and outer ear surfaces. During the challenge phase, on days 10, 13, 17, and 20, 100 μL of 0.5% DNCB solution (in the same vehicle) was applied to the skin site or 10 μL of 0.2% DNCB solution was applied to the ear site. The terminal experiment was performed on day 20, 1 h after the last oral or topical application of WTM. Mice were euthanized via CO_2_ anesthesia, and auricle thickness was measured. Full-thickness dorsal skin and ear tissues were collected for histological staining and pathological analysis. The wet weight of the spleen was also precisely measured (Figure 1A). The spleen index, an indicator of immune organ enlargement, was determined by calculating the ratio of spleen weight to total body weight and expressing the result as a percentage: (spleen weight/body weight) × 100%.

### 4.6. Evaluation of AD Severity Scoring

Dermatitis severity was evaluated by examining clinical features, including erythema, erosion, dryness, and lichenification, each graded according to the extent of affected skin area: 0 for no involvement, 1 for <25%, 2 for 25–50%, and 3 for >50%. The total dermatitis score (SCORAD) was obtained by summing the individual grades, yielding a maximum score of 12. Scoring was conducted by a pathologist unaware of the group allocation [44].

### 4.7. Scratching Behavior Test

To evaluate pruritus-related behavior, mice were monitored for scratching following the final DNCB challenge on day 20. After 1 h acclimation in the observation chamber, a 20 min video recording was conducted using Logitech Webcam Software (Version 2.10). Scratching episodes—defined by repetitive hind paw motion away from and back to the floor—were timed in seconds. To ensure reliability, all behavioral data were analyzed by the same investigator under blinded conditions, with both animals and recordings coded to eliminate bias [45].

### 4.8. Histological Examination and Scores

Following euthanasia, dorsal skin samples were collected using a sterile dissection kit. The tissues were fixed in 10% buffered formalin and processed for 14 h in an automated tissue processor (Leica Instrument GmbH, Wetzlar, Germany). Samples were subsequently embedded in paraffin, sectioned at 5 µm thickness, and stained with hematoxylin and eosin (H&E) for analysis. Epidermal thickness and eosinophil infiltration were evaluated using a compound microscope and Leica Application Suite Core (LAS) software (Version 4.12, Leica Microsystems GmbH, Wetzlar, Germany). Thickness measurements were conducted in five fields of the thickest epidermal area at 100× magnification in a double-blinded manner, while eosinophil counts were performed in ten randomly selected high-power fields at 400× magnification. A blue toluidine stain was used to examine the mast cells. The number of mast and degranulated mast cells was counted in ten random high-power fields at ×400 and ×1000 magnifications, respectively.

### 4.9. Serum Cytokine Level Assessment and IgE Assay

To evaluate inflammatory responses, serum levels of IL-4, IL-6, IL-1β, TNF-α, and IL-31 were measured using a multiplex cytokine assay kit (BioLegend, San Diego, CA, USA). In parallel, IgE levels were determined using a mouse-specific ELISA kit, also from BioLegend, according to the provided instructions. The absorbance of each sample was measured at a wavelength of 450 nm.

### 4.10. The TBARS (Thiobarbituric Acid Reactive Substance) Assay

Malondialdehyde (MDA) levels were assessed by investigating the thiobarbituric acid (TBA) reaction [46]. Renal tissue homogenization was performed with lysis buffer at 4 °C, followed by the addition of TBA solution to each sample. Incubation at 90 °C for 45 min facilitated the formation of the MDA-TBA adduct. The reaction mixture’s optical density (OD) was assessed by measuring absorbance at 532 nm, with TBARS levels subsequently calculated and reported as nmol per milligram of protein.

### 4.11. Glutathione (GSH) Asaay

Reduced GSH was quantified using the DTNB assay. Kidney tissues were first homogenized in ice-cold 10% trichloroacetic acid. Homogenates were centrifuged at 1500× *g* for 10 min at 4 °C to obtain the supernatant. This supernatant was then mixed with 0.1 M phosphate buffer (pH 8.4) and DTNB reagent. The absorbance was measured at 412 nm, and GSH concentrations were calculated from a standard curve prepared using known values [47].

### 4.12. Western Blot Analysis

Proteins were isolated from skin tissues via lysis buffer, and their concentrations were assessed using a commercial assay kit (Bio-Rad Laboratories, Inc., Hercules, CA, USA). After denaturation, samples (50 μg) underwent electrophoresis on a 12% SDS-PAGE gel and were transferred to PVDF membranes. Immunoblotting was performed by sequential incubation with primary and secondary antibodies. Signals were developed with an ECL detection system (Amersham International plc., Little Chalfont, Buckinghamshire, UK) and analyzed using ImageJ software (v1.x), while β-actin ensured normalization across experiments.

### 4.13. Statistical Analysis

Statistical analysis was conducted using SPSS software version 22.0 (SPSS, Inc., Chicago, IL, USA). Data were expressed as mean ± standard error of the mean (SEM). For comparisons between two groups, Student’s *t*-test was employed, while one-way ANOVA followed by Scheffe’s post hoc test was used for multiple group comparisons. A *p*-value less than 0.05 was considered statistically significant.

## 5. Conclusions

In conclusion, this study demonstrated that both topical and oral WTM administration effectively alleviated DNCB-induced AD-like skin inflammation in mice. These therapeutic effects were associated with reduced immune cell infiltration and decreased proinflammatory cytokine expression, including IL-4, IL-6, IL-1β, TNF-α, and IL-31. WTM also significantly suppressed histopathological changes and downregulated key inflammatory signaling pathways, namely, TLR4/NF-κB, MAPK, and JAK/STAT/TSLP. Phytochemical analysis revealed that chlorogenic, caffeic, and cichoric acids are the major active components of WTM. The observed immunomodulatory effects are likely due to the synergistic actions of these bioactive polyphenols with known anti-inflammatory and anti-allergic properties. Collectively, these findings support the potential of WTM as a promising pharmacological candidate for managing atopic dermatitis. In light of its dual-route efficacy and favorable safety profile, WTM may be further developed as a complementary or alternative therapeutic agent for AD, particularly for patients seeking plant-based or steroid-sparing treatments. Future translational studies and clinical trials will be essential to validate its therapeutic potential, optimize formulation strategies, and explore its integration into dermatological care regimens.

## Data Availability

Data are contained within the article.
